# Apple pomace addition improves fermentation quality, nutritive value and bacterial community succession of alfalfa silage

**DOI:** 10.5713/ab.260566

**Published:** 2026-09-01

**Authors:** Wei Wang, Minglu Zuo, Xiao Xiao, Le Wang, Jiayi Han, Siran Wang, Tianrong Zhou

**Affiliations:** 1 Key Laboratory of Innovative Research and Application of Native Plant Germplasm Resources of Hulunbuir, College of Agriculture, HulunBuir University, HulunBuir, China; 2 Institute of Animal Science, Jiangsu Academy of Agricultural Sciences, Nanjing, China; 3 Key Laboratory for Crop and Animal Integrated Farming, Ministry of Agriculture and Rural Affairs, Nanjing, China

**Keywords:** Alfalfa, Apple Pomace, Bacterial Community, By-product Utilization, Silage Quality

## Abstract

**Objective:**

Apple pomace is an abundant by-product high in soluble carbohydrates, but its ability to improve alfalfa silage fermentation remains underexplored. This study aimed to investigate the effects of adding apple pomace addition on the nutritional composition, fermentation quality, and bacterial community succession of alfalfa silage.

**Methods:**

Alfalfa (357 g/kg fresh weight [FW]) was harvested at the early flowering stage and ensiled with no apple pomace (CK), 5% FW apple pomace (AL), and 10% FW apple pomace (AH) for 60 days at room temperature (23±1°C). The nutritional content, fermentation quality, and bacterial community were examined.

**Results:**

AH significantly improved silage quality compared with CK, evidenced by higher (p<0.05) lactic acid (28.41 vs. 15.24 g/kg dry matter [DM]), acetic acid (6.8 vs. 2.3 g/kg DM), DM (498 vs. 358 g/kg fresh matter), crude protein (193 vs. 154 g/kg DM), and water-soluble carbohydrates (57.7 vs. 45.1 g/kg DM), and lower (p<0.05) pH (3.75 vs. 4.81) and ammonia nitrogen (38.4 vs. 69.8 g/kg TN). At the phylum level, Pseudomonadota (35.94%) and Bacillota (64.06%) dominated CK, whereas apple pomace supplementation increased Bacillota to 87.78% and decreased Pseudomonadota to 3.93%. At the genus level, apple pomace boosted *Weissella*, *Lactiplantibacillus*, and *Bacillus*. AL was dominated by *Weissella* (42.65%) and *Bacillus* (95.91%), while AH was predominantly colonized by *Weissella* (38.89%), *Lactiplantibacillus* (20.04%), *Levilactobacillus* (10.53%), and *Lacticaseibacillus* (8.79%), forming a more diverse lactic acid bacteria consortium. Spearman correlation analysis showed that *Lactiplantibacillus* and *Weissella* were positively correlated (p<0.05) with lactic acid and nutritional parameters, and negatively correlated (p<0.05) with pH. Functional profiling indicated that apple pomace reshaped metabolic functions and upregulated enzymes related to carbohydrate degradation, energy metabolism, and substrate transport, promoting lactic acid accumulation.

**Conclusion:**

Apple pomace improved fermentation performance and nutrient retention in alfalfa silage, and a 10% inclusion rate is recommended for practical production.

## INTRODUCTION

Apple pomace is the typical by-product of the apple processing industry, and its global yield exceeds 3,600 kilotons per year. It is rich in crude protein (CP; 6.0–7.0 g/100 g dry matter [DM]), water soluble sugars (33.0–38.0 g/100 g DM), organic acids (2.5–4.5 g/100 g DM), vitamins, minerals, and pectin (14.5–18.5 g/100 g DM). Fresh apple pomace has a high moisture content of 70%–85%, which hinders its preservation and direct feeding. Meanwhile, it features rich nutrients and good palatability, thus serving as a valuable unconventional feed resource. However, most apple pomace is currently discarded directly as industrial waste, which not only causes severe environmental pollution but also results in a considerable waste of biological resources [3]. Therefore, the efficient utilization of apple pomace is of great significance to alleviating feed shortages, reducing production costs, and promoting the recycling of agricultural by-products [4].

To date, the primary utilization approaches for apple pomace include drying, fresh feeding, ensiling, and microbial fermentation [5]. Among these strategies, co-ensiling apple pomace with alfalfa is a promising technical route to address the inherent drawbacks of single raw materials. Apple pomace contains abundant water-soluble carbohydrates (WSCs), which can compensate for the insufficient carbon sources in alfalfa. In turn, alfalfa offsets the excess moisture and crude fiber content of apple pomace. Such mixed ensiling ultimately improves fermentation quality and prolongs the storage life of the two forage materials [6]. Nevertheless, apple pomace has inherent limitations, such as high levels of tannins, which limit its direct use in production [7]. The type of pomace applied determined some variations, but it was observed that at a highest level of tannins (385.26 mg GAE/100 g) [8]. Previous studies have demonstrated that blending apple pomace with high-fiber or high-protein forages and supplementing it with lactic acid bacteria (LAB) can optimize fermentation conditions, accelerate the decline in pH, inhibit harmful microorganisms, and reduce protein degradation [8]. Most previous studies primarily concentrated on the fermentation quality of silage prepared with single apple pomace or its combinations with conventional forages. However, few efforts have been devoted to exploring how apple pomace supplementation modulates the bacterial community structure during alfalfa ensiling, and the underlying microbial mechanisms remain poorly understood.

Alfalfa is a high-quality legume forage widely used in ruminant production; however, its low WSC content (2.5%–5.0% DM) frequently leads to inferior fermentation quality thereby limiting its large-scale application [9]. Apple pomace is rich in soluble carbohydrates, which can provide sufficient fermentable substrates for LAB, thereby promote lactic acid fermentation, and improve the quality of alfalfa silage [10]. Practically, the addition dosage plays a decisive role in silage production. A low proportion of apple pomace cannot optimize the fermentation process of alfalfa silage. By contrast, excessive addition will change the nutritional balance, suppress the proliferation of functional bacteria, break the stability of microbial community, and ultimately lead to poor fermentation quality [11].

Therefore, this study aimed to evaluate the effects of apple pomace addition on alfalfa silage fermentation quality, nutritive value and bacterial community succession, and identify the optimal inclusion rate. The results could provide a theoretical basis and technical support for the high-value utilization of apple pomace and the production of high-quality alfalfa silage.

## MATERIALS AND METHODS

### Experimental materials and silage preparation

Alfalfa (cv. Challenger) was grown at the Dongshan Campus of Hulunbuir University and harvested at the early flowering stage. Apple pomace was prepared from defective apples purchased from Lidong Supermarket in Hulunbuir City. The apples were crushed into pomace using a Supor grinder (JR05-300) and then air-dried before use. Alfalfa was wilted to a target DM content of approximately 350 g/kg fresh matter (FM), and then chopped into 1–2 cm segments using a forage cutter. *Lactiplantibacillus plantarum* was added at 1×10^6^ CFU/g FM (LAB≥2.0×10^10^ CFU/g, purchased from Shanghai Macklin Biochemical).

Three treatments were established:

CK: control (no additive)
AL: 5% apple pomace (based on fresh alfalfa weight)
AH: 10% apple pomace (based on fresh alfalfa weight)

The replacement ratios of apple pomace were set at 5% and 10% (All apple pomace supplementation ratios in this study are defined based on the fresh weight of alfalfa) in the present study. These gradient levels were determined with reference to previous relevant studies on apple pomace application in forage silage and total mixed ration silage, aiming to explore the dose-dependent effects of apple pomace substitution on fermentation characteristics, nutrient digestibility and ruminal fermentation parameters [12]. Consistent *Lactiplantibacillus* inoculation treatment was applied to all three experimental groups with the same inoculation time and dosage. The final viable bacterial concentration of each group reached the stated CFU/g, which ensured unified initial microbial conditions among treatments and eliminated experimental bias caused by inconsistent inoculation. The silage materials were packed into plastic bags at 500 g per bag, with six replicates per treatment. All six silage bags were sampled and analyzed independently in this study. No samples were pooled or mixed during the whole experimental detection process. All data represent independent biological replicates to ensure the authenticity and reliability of experimental data. All bags were then compacted, vacuum-sealed, labeled, and stored at room temperature (20±1°C). After 60 d of ensiling, the silage bags were opened and sampled for the analysis of chemical composition, fermentation characteristics, and microbial communities.

### Sample collection and chemical analysis

Silage samples were dried in a forced-air oven at 65°C for 48 h to determine DM content. Dried samples were ground and passed through a 1 mm sieve for chemical analyses. CP content was measured using the Kjeldahl method. Neutral detergent fiber (NDF) and acid detergent fiber (ADF) contents were determined using the Van Soest method with an ANKOM 200 Fiber Analyzer (ANKOM Technology), as described by [13] with minor modifications. Briefly, approximately 1.0 g of ground sample (passed through a 1 mm sieve) was weighed into a pre-dried and pre-weighed ANKOM F57 filter bag, heat-sealed, and subjected to reflux with neutral detergent solution (containing sodium lauryl sulfate, EDTA disodium salt, sodium tetraborate decahydrate, disodium hydrogen phosphate, and 2-ethoxyethanol, pH 6.9–7.1) at 100°C for 60 min; after refluxing, the bags were rinsed three times with hot water (90°C–100°C) and once with acetone, oven-dried at 105°C for 4 h to constant weight, cooled in a desiccator, and weighed to calculate NDF content based on the residue weight corrected for a blank bag. The NDF residue in the filter bag was then refluxed with acid detergent solution (containing cetyltrimethylammonium bromide in 1.00 mol/L sulfuric acid) at 100°C for 60 min, followed by the same rinsing, acetone washing, drying, cooling, and weighing procedures as for NDF, and ADF content was calculated similarly based on the acid detergent-treated residue weight; all analyses were performed in duplicate, and results were expressed as g/kg of DM. WSC were determined using the anthrone-sulfuric acid colorimetric method. All analyses were performed in triplicate.

Silage extracts were prepared by mixing 20 g of silage sample with 180 mL of distilled water. The mixture was incubated at 4°C for 24 h and then filtered through four layers of cheesecloth to obtain the filtrate for subsequent analysis. The pH value was measured using an S470 pH meter (Mettler Toledo). Lactic acid, acetic acid, propionic acid and butyric acid were analyzed via high-performance liquid chromatography equipped with a KC-811 column (Shimadzu). The column temperature was maintained at 50°C, the flow rate was 1 mL/min, and the detection wavelength of the SPD detector was set to 210 nm. Ammonia nitrogen content was determined using the phenol-hypochlorite colorimetric method according to [14].

### Analysis of bacterial community composition

Total microbial genomic DNA was extracted from silage specimens with a commercial DNA extraction kit. The concentration and purity of recovered DNA were determined using a NanoDrop spectrophotometer. The V3–V4 hypervariable segment of the bacterial 16S rRNA gene was amplified using the universal primer set 338F/806R for bacterial community profiling. Valid amplicons were subsequently sequenced on the Illumina MiSeq platform. Raw sequencing reads underwent quality trimming and assembly, and the processed sequences were clustered into operational taxonomic units (OTUs) at a 97% similarity cutoff. Taxonomic assignment of bacterial OTUs was performed against the SILVA reference database. To facilitate high-resolution bacterial taxonomic identification, full-length 16S rRNA gene sequencing was carried out via single-molecule real-time (SMRT) sequencing on the PacBio Sequel II platform. Bioinformatic workflows including quality control, sequence clustering, taxonomic annotation and microbial diversity evaluation were completed using QIIME2 and R software [15].

### Functional prediction analysis

Functional potential of the bacterial communities was predicted using Phylogenetic Investigation of Communities by Reconstruction of Unobserved States (PICRUSt2) based on 16S rRNA gene sequences. Predicted metagenomes were analyzed for Kyoto Encyclopedia of Genes and Genomes (KEGG) orthologs. Additionally, BugBase was used to predict phenotypic traits, including Gram staining, oxygen tolerance, and biofilm formation, among others.

### Statistical analyses

Data on chemical composition and fermentation parameters were analyzed using one-way analysis of variance (ANOVA) in SPSS 22.0. Means were compared by Duncan’s multiple range test, with significance defined at p<0.05.

## RESULTS

### Effects of apple pomace addition on chemical composition of alfalfa silage

The chemical composition of raw alfalfa and apple pomace materials is presented (Table 1). Both raw materials possessed suitable pH values for ensiling, with sufficient basic nutritional components for silage preparation. The chemical composition of alfalfa silage after 60 days of fermentation is shown (Table 2). Apple pomace supplementation significantly altered the nutritional profiles of alfalfa silage, with distinct dose-dependent differences observed among the CK, AL, and AH treatments.

Compared with the CK group, both AL and AH groups exhibited significantly improved nutritional quality, with higher contents of DM, CP, NDF, ADF, and WSC. Further comparison between treatment groups showed that the AH group (10% apple pomace) obtained superior nutrient retention. Specifically, the DM (498 vs. 436 g/kg FM), CP (193 vs. 173 g/kg DM), and WSC (57.7 vs. 51.2 g/kg DM) contents in AH were significantly higher than those in the AL group (5% apple pomace). Linear and quadratic analyses further verified the dose effects of apple pomace supplementation. All nutritional indicators showed significant linear increasing trends with the elevation of apple pomace addition. DM, NDF, and ADF presented significant quadratic responses, while CP and WSC exhibited stable linear growth without remarkable quadratic variation, indicating that increased apple pomace supplementation effectively promoted nutrient accumulation in alfalfa silage within the experimental gradient.

### Effects of apple pomace addition on fermentation quality of alfalfa silage

The fermentation quality of alfalfa silage was significantly optimized by apple pomace supplementation in a dose-dependent manner (Table 3). Compared with CK, AL and AH groups showed markedly reduced pH and ammonia nitrogen content, as well as significantly increased lactic acid and acetic acid accumulation, reflecting improved fermentation performance. No propionic acid or butyric acid was detected in any treatment, indicating that all silage groups achieved favorable fermentation status without undesirable spoilage.

Among the three silage treatments, the AH group showed the best fermentation characteristics. The pH value decreased sequentially from CK (4.81) to AL (4.12) and AH (3.75), while lactic acid content increased gradually from 15.2 g/kg DM (CK) to 25.7 g/kg DM (AL) and 28.4 g/kg DM (AH). Meanwhile, the AH group had higher acetic acid content (6.87 g/kg DM) than the AL group (5.12 g/kg DM) and a significantly lower ammonia nitrogen ratio. Linear and quadratic analyses confirmed that pH, lactic acid, acetic acid, and ammonia nitrogen were all significantly affected by apple pomace dosage, showing regular linear changes and curvilinear variation trends. These results fully demonstrated that appropriate apple pomace addition effectively promoted lactic acid fermentation, inhibited harmful fermentation, and improved the overall fermentation quality of alfalfa silage, with the 10% addition level achieving the optimal effect.

### Effects of apple pomace addition on bacterial community diversity and composition

The bacterial community structure and diversity of ensiled alfalfa were strongly modulated by apple pomace supplementation, with obvious differences in microbial succession characteristics among CK, AL, and AH groups. Notably, the higher microbial richness and diversity in FM is an inherent ecological characteristic of aerobic fresh forage epiphytic microbiota, which contains abundant unfiltered environmental microorganisms. This higher diversity does not indicate superior fermentation quality, as complex epiphytic communities cannot survive and function under anaerobic and acidic silage conditions.

At the phylum level, the silage bacterial communities of all three treatments were consistently dominated by Bacillota, with relative abundances ranging from 64% to 88% (Figure 1C). In contrast, the non-ensiled FM group was dominated by Pseudomonadota and Actinomycetota, which were rarely enriched in fermented silage. Focusing on silage treatment comparisons, apple pomace supplementation further increased the relative abundance of Bacillota and eliminated aerobic and acid-sensitive phyla, simplifying the microbial community structure toward a fermentation-adapted profile.

At the genus level, distinct community differentiation was observed among CK, AL, and AH groups (Figure 1D). The CK group was dominated by *unclassified Lachnospiraceae*, while the two apple pomace-treated groups were dominated by beneficial LAB. Weissella was the predominant genus in both AL and AH groups, serving as the core functional microbe driving silage acidification. Importantly, dosage-dependent differences were clearly identified between treatment groups: the AL group relied mainly on *Weissella* and *Bacillus*, whereas the AH group formed a more diverse and stable lactic acid bacterial consortium, with significantly enriched *Lactiplantibacillus*, *Levilactobacillus*, and *Lacticaseibacillus*. This diversified functional flora enabled the high-dose apple pomace group to achieve better fermentation stability.

Alpha diversity analysis further confirmed the microbial succession pattern during ensiling (Figure 2). The FM group exhibited higher richness and diversity, which was attributed to the complex aerobic epiphytic microbiota of fresh forage. After anaerobic fermentation, microbial diversity of all silage groups decreased significantly, and the community tended to be specialized and stable. No excessive redundant microbial taxa were retained in CK, AL, and AH, and the community variation was mainly driven by apple pomace dosage rather than initial raw material microbiota.

Principal coordinate analysis (PCoA) and PERMANOVA analysis based on genus-level community data revealed significant microbial structural differentiation among the three silage treatments (R = 0.9167, p = 0.001) (Figure 3). The AH group formed an independent cluster clearly separated from AL and CK, further verifying that apple pomace supplementation induced dose-dependent shifts in bacterial community composition and successfully reshaped the silage microecology.

### Linear discriminant analysis effect size differential species analysis and multiple-group comparison

Linear discriminant analysis effect size (LEfSe) analysis screened specific microbial biomarkers for each silage treatment group, clarifying the regulatory effect of apple pomace on functional flora enrichment (Figure 4). The CK group was characterized by unbeneficial spoilage-related taxa (*Lachnospiraceae* and *Enterobacterales*). In contrast, apple pomace supplementation specifically enriched fermentation-beneficial Bacillota-affiliated genera. The AL group was uniquely characterized by Weissella and Bacillus, while the AH group was distinguished by the co-enrichment of multiple high-efficiency LAB, including *Weissella*, *Lactiplantibacillus*, *Levilactobacillus*, and *Lacticaseibacillus*. Kruskal-Wallis H testing further verified these inter-group differences (Figure 5). The cooperative enrichment of multiple functional LAB in the AH group explained its superior fermentation and nutrient retention performance compared with the low-dose treatment.

Genus-level heatmap analysis intuitively reflected the community clustering characteristics of each treatment (Figure 6). The core functional LAB (*Weissella*, *Lactiplantibacillus*) were highly enriched in AL and AH groups, whereas acid-sensitive taxa dominant in FM were almost eliminated in fermented silage. The consistent clustering of AL and AH and their clear separation from CK confirmed that apple pomace addition was the core factor driving the enrichment of beneficial functional flora in alfalfa silage.

### Correlation analysis between bacterial communities and physicochemical parameters

Spearman correlation analysis clarified the internal relationship between core microbial community variation and silage quality improvement (Figure 7). The dominant functional LAB in apple pomace-treated groups, including *Weissella*, *Lactiplantibacillus*, and *Lacticaseibacillus*, were positively correlated with key nutritional indicators (DM, CP, WSC, NDF, ADF) and negatively correlated with pH. In contrast, acid-sensitive taxa such as *Massilia* showed opposite correlation patterns. These results indicated that the dose-dependent enrichment of beneficial LAB induced by apple pomace supplementation effectively promoted silage acidification and nutrient retention, while inhibiting the growth of unfavorable microorganisms, ultimately improving the overall quality of alfalfa silage.

### Functional prediction analysis (PICRUSt2)

PICRUSt2 functional prediction was performed to preliminarily explore the metabolic variation of microbial communities in different silage treatments. The enzyme abundance profiles differed significantly among CK, AL, and AH groups, reflecting distinct microbial metabolic characteristics under different apple pomace dosages (Figure 8). Overall, enzymes related to nucleic acid metabolism and protein synthesis were more abundant in the CK group, while apple pomace treatment groups (AL and AH) exhibited higher enrichment of carbohydrate degradation and substrate transport-related enzymes, including 6-phospho-beta-glucosidase and acetyl-CoA carboxylase.

The reduced abundance of nucleic acid replication and protein synthesis-related enzymes in AL and AH groups mainly reflected the inhibited proliferation of undesirable acid-sensitive microorganisms under acidic fermentation conditions. The suppression of miscellaneous microbial metabolism reduced nutrient competition and ineffective consumption during ensiling. Meanwhile, the retention and metabolic activity of beneficial LAB were guaranteed, which promoted efficient carbohydrate degradation and lactic acid accumulation. These functional changes further illustrated that apple pomace supplementation optimized microbial metabolic patterns, eliminated invalid microbial metabolism, and facilitated the formation of high-quality silage fermentation microenvironment.

## DISCUSSION

The present study evaluated the effects of gradient apple pomace supplementation (0%, 5%, and 10% fresh weight) on the fermentation quality, nutritional value, and bacterial community succession of alfalfa silage, with a clear focus on treatment differences among CK, AL, and AH groups. The results demonstrated that apple pomace addition significantly improved silage nutrient retention and fermentation performance. Specifically, supplemental apple pomace increased DM, CP, and WSC contents, lowered pH and ammonia nitrogen accumulation, and promoted lactic and acetic acid accumulation [16,17]. No undesirable propionic or butyric acid was detected in any treatment, indicating well-preserved silage fermentation [18]. These improvements were mainly attributed to the high level of soluble carbohydrates in apple pomace, which compensated for the insufficient fermentable substrates in alfalfa and provided continuous carbon sources for lactic acid bacterial proliferation and acid production during ensiling [19,20]. Similarly, Zhang et al. [21] demonstrated that integrated organic-inorganic fertilization could enhance rice straw productivity and silage quality by improving the fermentable substrate availability.

Apple pomace supplementation strongly reshaped the bacterial community structure of alfalfa silage and drove the succession toward a LAB-dominated microbial ecosystem [22]. Consistent with this, Chen et al. [23] found that co-ensiling whole-plant mulberry with navel orange residue not only improved fermentation quality but also significantly modulated bacterial community composition, highlighting the role of fruit by-products in reshaping silage microecology. The higher microbial diversity observed in fresh alfalfa material is an inherent characteristic of aerobic forage epiphytic microbiota and does not represent superior fermentation quality. During anaerobic ensiling, continuous acidification significantly suppressed acid-sensitive epiphytic microorganisms such as *Massilia* and *Sphingomonas*, resulting in a general decline in their abundance across all silage groups. In contrast, acid-tolerant beneficial LAB became progressively dominant under anaerobic acidic conditions. Focusing on silage treatment comparisons, apple pomace addition selectively enriched core functional LAB taxa [24]. The genus *Weissella* dominated both apple pomace-treated groups and contributed to rapid acidification and inhibition of spoilage microorganisms [25]. Notably, the 10% apple pomace group (AH) established a more diverse and stable LAB consortium, with significantly higher abundances of *Lactiplantibacillus*, *Levilactobacillus*, and *Lacticaseibacillus* compared with the 5% addition group (AL). This diversified LAB community further stabilized the silage microenvironment and improved nutrient preservation, explaining the better fermentation performance observed in the AH treatment.

PCoA and PERMANOVA analyses confirmed distinct bacterial community structures among CK, AL, and AH groups, demonstrating that apple pomace dosage was a key factor modulating microbial succession during ensiling [26]. Similarly, Zhang et al. [27] reported that optimizing additive combinations in peanut vine silage effectively improved fermentation quality and feed efficiency, further supporting the idea that targeted supplementation can steer microbial community succession toward a beneficial direction. Consistent with community variation, LEfSe analysis identified unique LAB biomarkers in apple pomace-supplemented groups, further verifying that apple pomace effectively screens beneficial microorganisms and optimizes silage microecology [28]. Spearman correlation analysis revealed close associations between core microbial genera and silage physicochemical properties. Key functional LAB including *Weissella*, *Lactiplantibacillus*, and *Lacticaseibacillus* were positively correlated with nutritional indicators and negatively correlated with pH, confirming their vital roles in improving fermentation quality and nutrient retention [29].

PICRUSt2 functional prediction was applied to preliminarily explore microbial metabolic potential during ensiling [30]. It is necessary to emphasize that PICRUSt2 results represent predicted functional profiles rather than direct in situ enzyme activity or gene expression measurements, and these predicted pathways only reflect correlational trends rather than definite causal relationships [31]. In the present study, the predicted reduction in nucleic acid replication and protein synthesis-related enzyme abundance in apple pomace-treated silage primarily reflected the inhibited proliferation of undesirable microorganisms under acidic stress [32–34]. This metabolic suppression of miscellaneous bacteria reduced microbial competition and nutrient consumption during ensiling. Importantly, this functional alteration at the microbial community level does not conflict with the increased CP content in silage. The elevated CP in apple pomace-treated groups was mainly attributed to reduced proteolysis and nutrient degradation, whereas the decreased protein synthesis pathway abundance corresponded to the suppressed growth of harmful microbes [35]. Overall, apple pomace addition optimized microbial community functions by inhibiting the metabolic activity of spoilage microorganisms while maintaining the dominance and metabolic activity of beneficial LAB, thereby facilitating high-quality silage fermentation [31].

In conclusion, under the experimental conditions of this study, 10% fresh-weight apple pomace supplementation achieved the optimal fermentation characteristics and nutritional quality of alfalfa silage. Apple pomace improved alfalfa silage performance by providing sufficient fermentable substrates for LAB growth, promoting rapid and stable acidification to inhibit spoilage microbes, and restructuring the bacterial community toward a fermentation-favorable functional profile. This study provides a feasible technical approach for the high-value utilization of apple pomace by-products and the production of high-quality alfalfa silage.

## CONCLUSION

Under the experimental conditions of the present study, the supplementation of 10% apple pomace exhibited the optimal fermentation characteristics and nutritional quality of alfalfa silage.

## Figures and Tables

**Figure 1:**
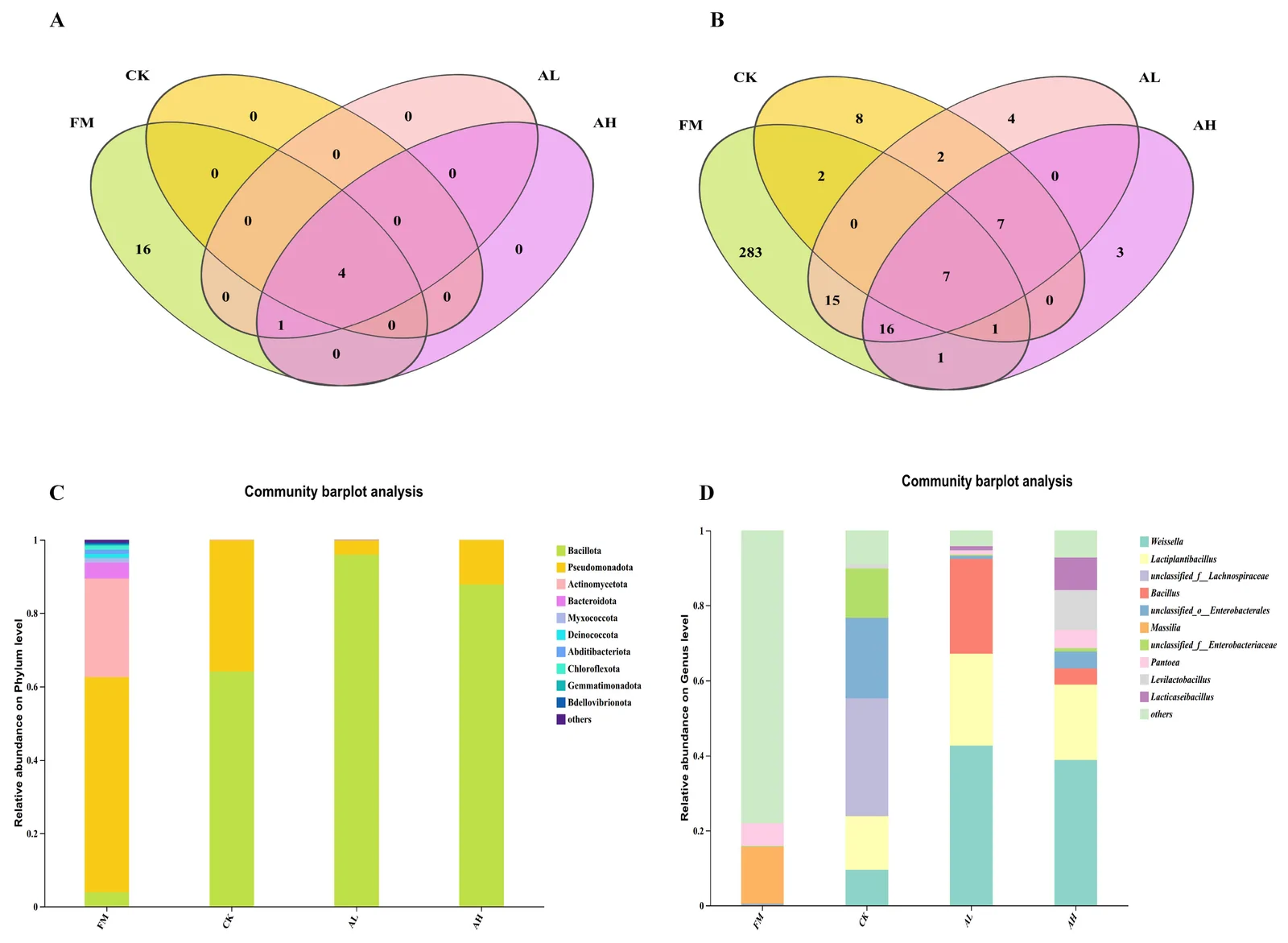
Effects of different apple pomace addition rates on the bacterial community composition of alfalfa silage. (A) Venn diagram showing shared and unique operational taxonomic units (OTUs) among treatments. (B) Venn diagram of genus-level OTUs. (C) Relative abundance of bacterial phyla. (D) Relative abundance of bacterial genera. Only phyla/genera with relative abundance >1% are shown; others are grouped as “Others”. Error bars (when applicable) represent standard deviation of three biological replicates. Statistical significance was determined by one-way ANOVA (p< 0.05). FM, fresh material before ensiling; CK, control (no apple pomace addition); AL, low apple pomace addition (5% fresh weight of alfalfa); AH, high apple pomace addition (10% fresh weight); ANOVA, analysis of variance.

**Figure 2:**
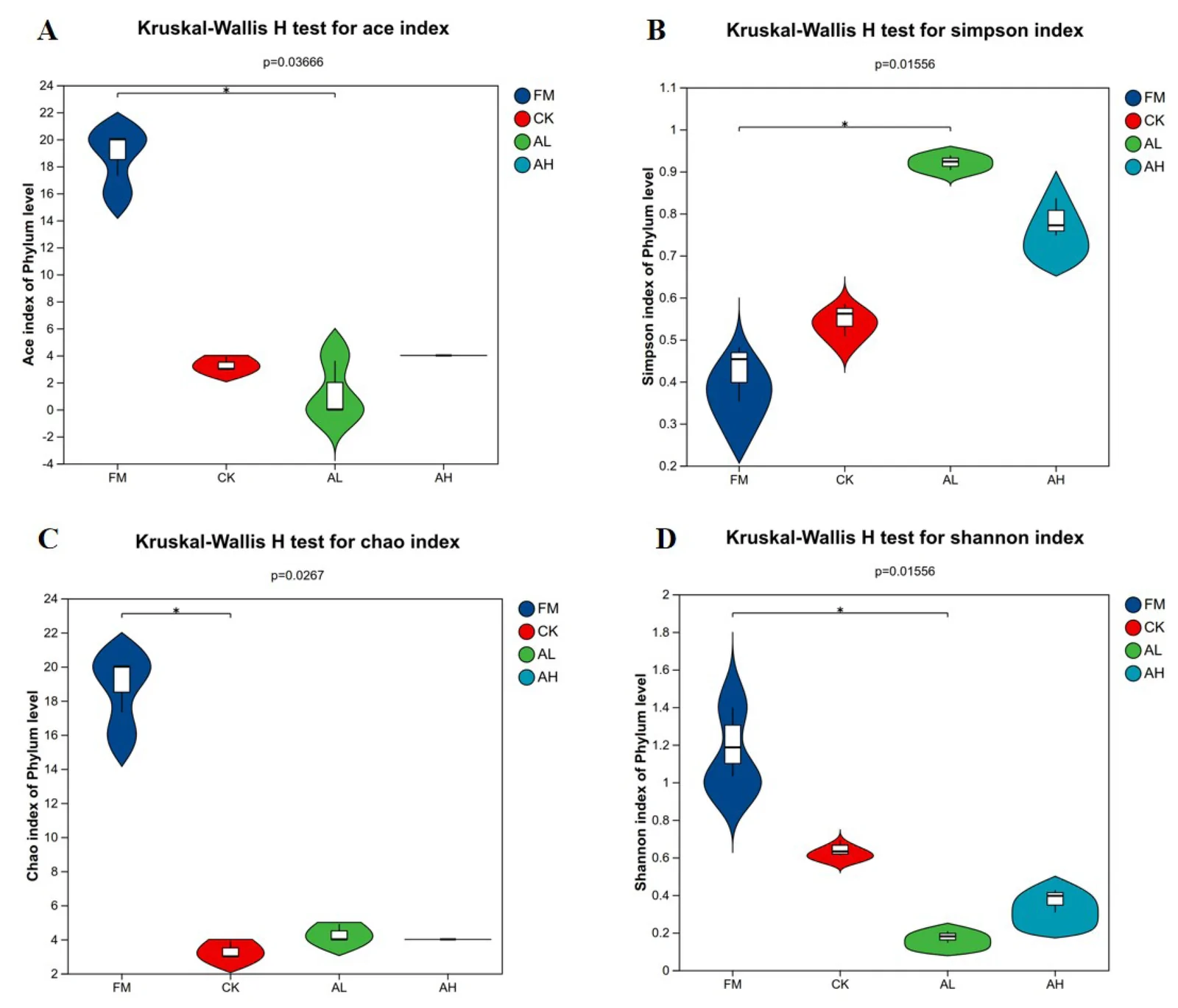
Kruskal-Wallis H test of bacterial community alpha diversity indices in alfalfa silage with different apple pomace addition rates. (A) ACE index, (B) Simpson index, (C) Chao index, (D) Shannon index. Alpha diversity indices were calculated based on operational taxonomic units (OTUs) clustered at 97% similarity from 16S rRNA gene sequences. Brackets with asterisks indicate significant differences between groups as determined by Kruskal-Wallis H test (* p<0.05), with exact p values shown above each panel. Boxes represent interquartile ranges, whiskers indicate 1.5× the interquartile range, and horizontal lines within boxes denote medians. Colors: FM (blue), CK (red), AL (green), AH (light blue). The analysis was performed using QIIME2. FM, fresh material before ensiling; CK, control (no apple pomace addition); AL, low apple pomace addition (5% fresh weight of alfalfa); AH, high apple pomace addition (10% fresh weight).

**Figure 3:**
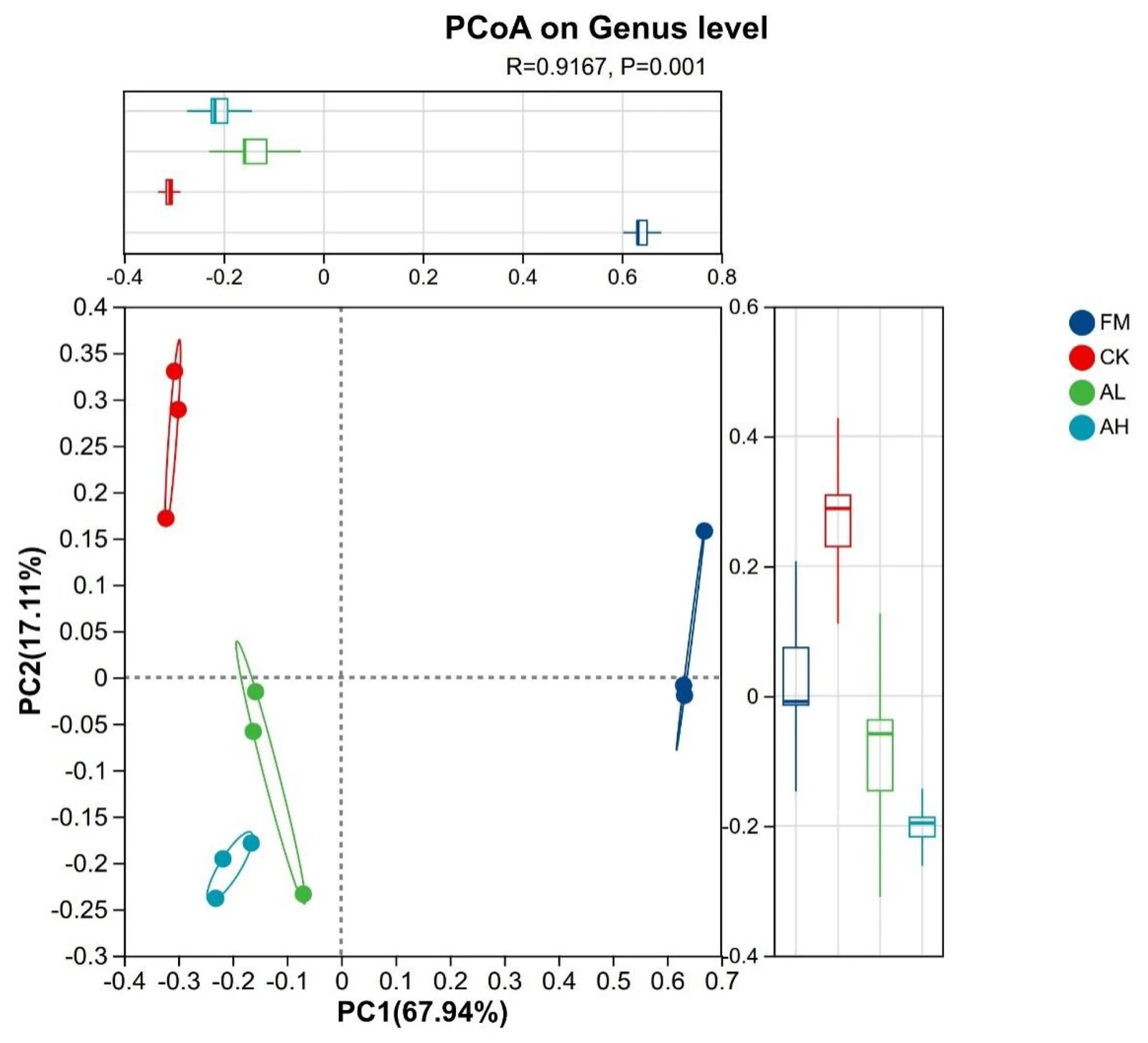
Genus-level principal coordinate analysis (PCoA) showing beta diversity of microbial communities. PCoA plot showing beta diversity of bacterial communities at the genus level among different treatments. PCoA was based on Bray-Curtis dissimilarity calculated from genus-level relative abundances. Each dot represents an individual silage sample (n = 6 per treatment). Colors: FM (blue), CK (red), AL (green), AH (light blue). Ellipses indicate 95% confidence intervals for each group. PERMANOVA (Adonis) was used to test for significant differences among groups (R^2^ = 0.9167, p = 0.001). The analysis was performed using R software with the vegan package. FM, fresh material before ensiling; CK, control (no apple pomace addition); AL, low apple pomace addition (5% fresh weight of alfalfa); AH, high apple pomace addition (10% fresh weight).

**Figure 4:**
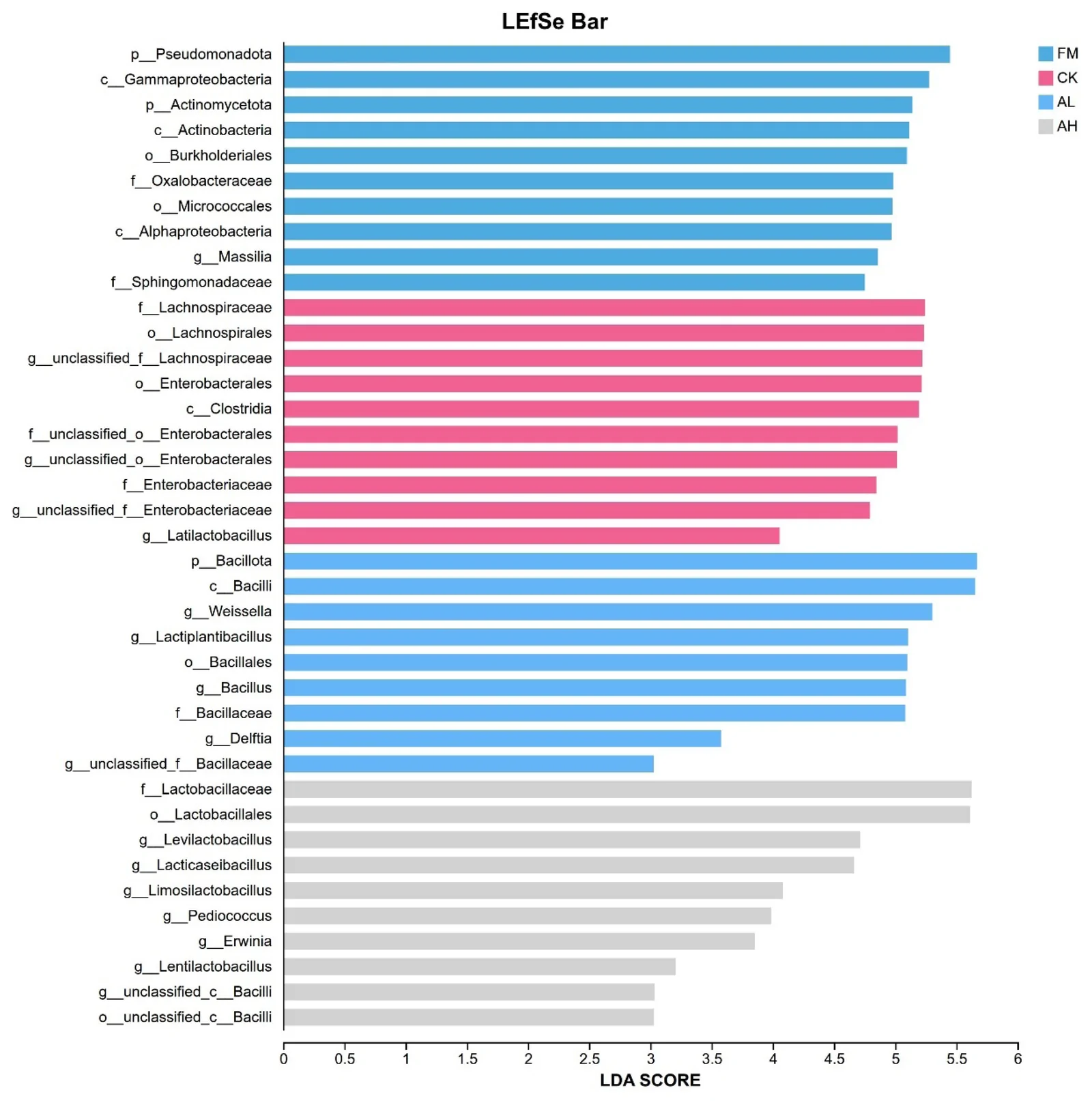
Linear discriminant analysis effect size (LEfSe) bar chart of differential microbial biomarkers. LEfSe bar chart of differentially abundant microbial biomarkers among treatments. LEfSe was used to identify taxa with significant differential abundance (LDA score>2, p<0.05) among FM, CK, AL, and AH groups. Only taxa with LDA scores above the threshold are shown. Colors indicate the treatment in which the taxon is enriched: blue (FM), magenta (CK), light blue (AL), gray (AH). The analysis combined the Kruskal-Wallis test for pairwise comparisons and linear discriminant analysis to estimate effect sizes. FM, fresh material before ensiling; CK, control (no apple pomace addition); AL, low apple pomace addition (5% fresh weight of alfalfa); AH, high apple pomace addition (10% fresh weight).

**Figure 5:**
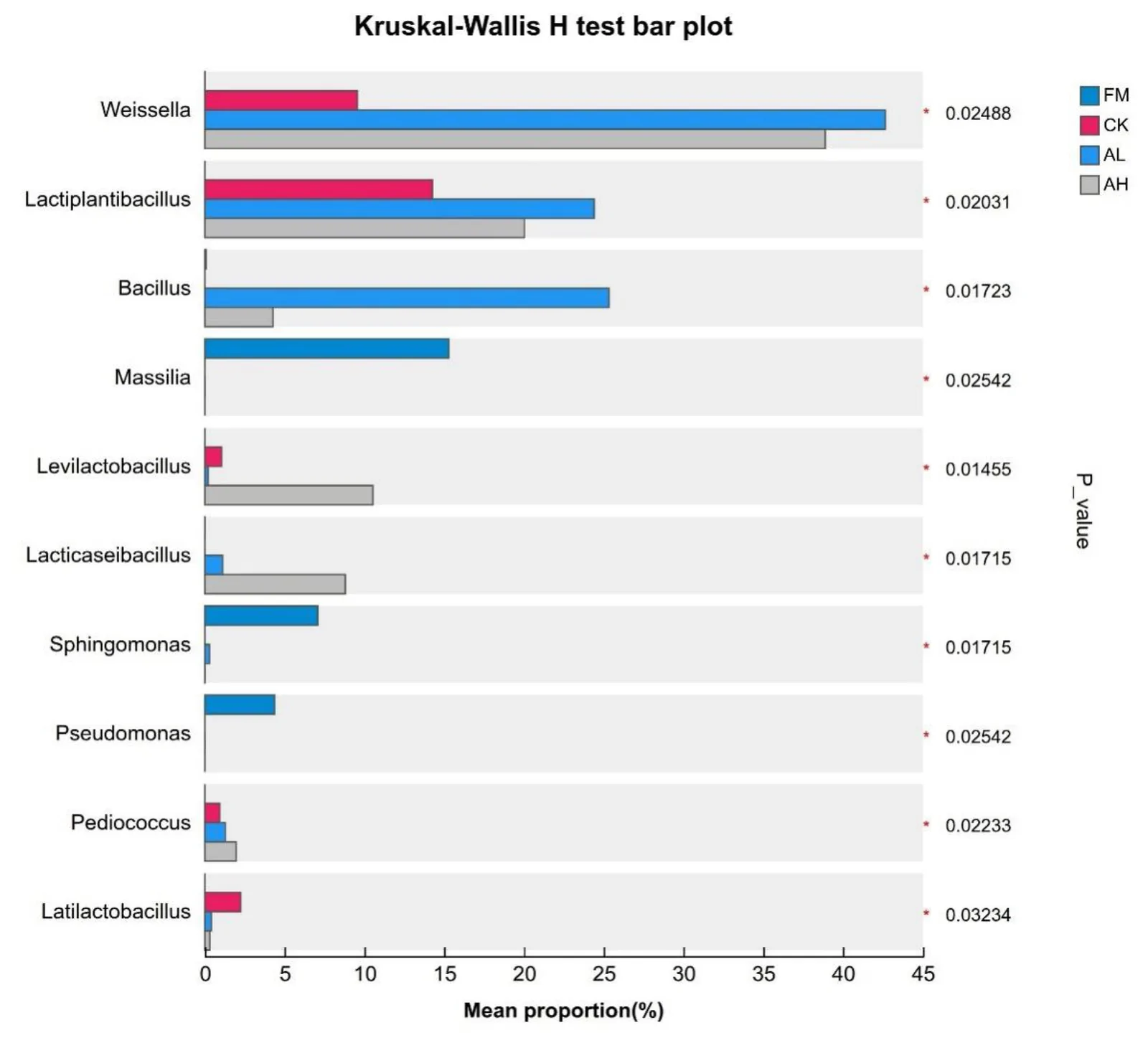
Multiple-group comparison of relative abundance of microbial species. Multiple-group comparison of relative abundances of key microbial genera among treatments. Bars represent mean relative abundance±standard error of the mean (SEM) for selected genera showing significant differences (Kruskal-Wallis test, p<0.05). Genera include *Weissella*, *Lactiplantibacillus*, *Bacillus*, *Massilia*, *Levilactobacillus*, *Lacticaseibacillus*, *Sphingomonas*, *Pseudomonas*, *Pediococcus*, and *Latilactobacillus*. Asterisks indicate significant differences among treatments (Kruskal-Wallis H test, * p<0.05), with exact p values shown to the right of each bar. Colors: FM (dark blue), CK (magenta), AL (light blue), AH (gray). FM, fresh material before ensiling; CK, control (no apple pomace addition); AL, low apple pomace addition (5% fresh weight of alfalfa); AH, high apple pomace addition (10% fresh weight).

**Figure 6:**
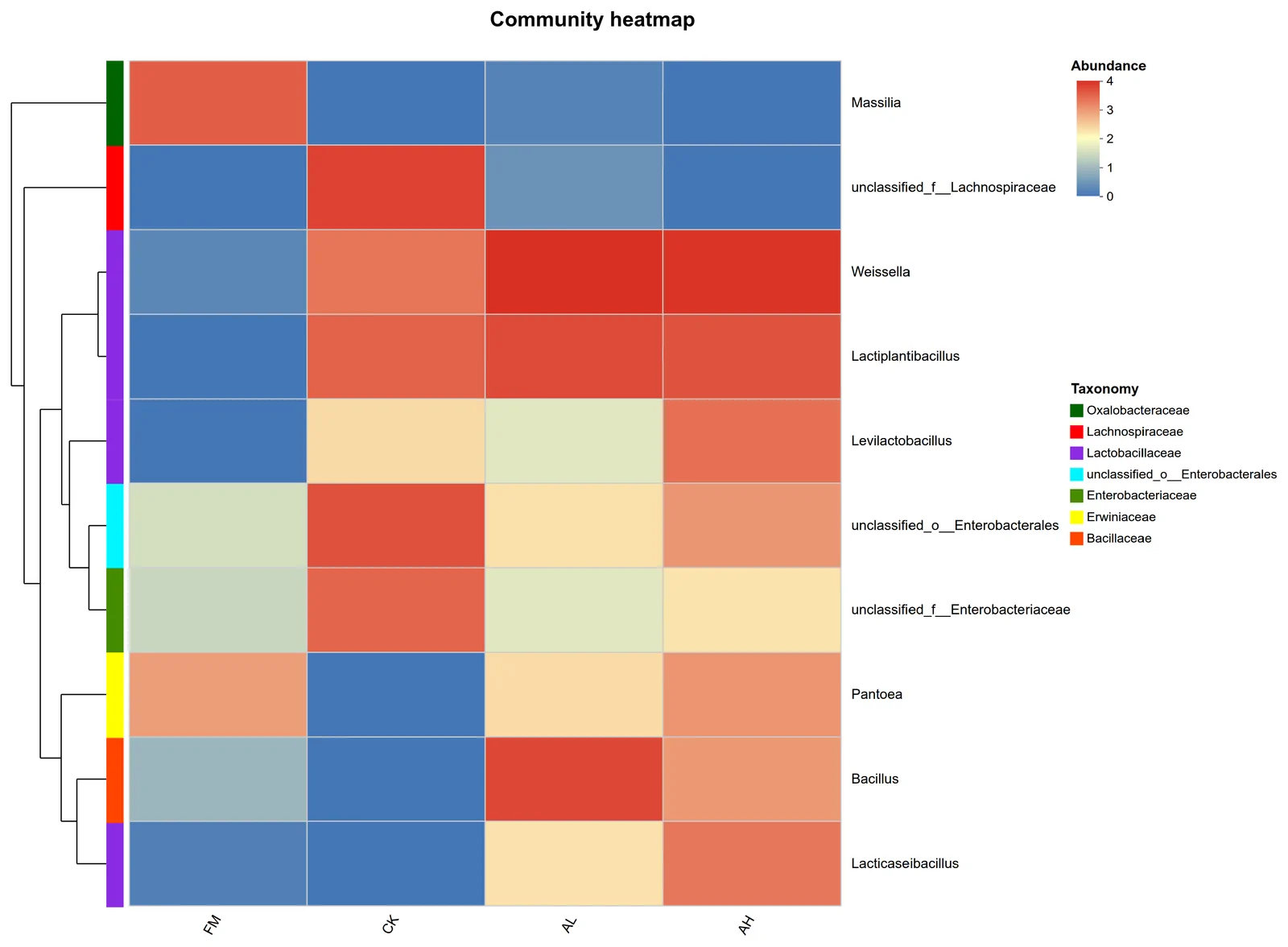
Heatmap of dominant microbial genus abundance. Heatmap of dominant bacterial genus abundance profiles across all samples. Rows represent the dominant bacterial genera, and columns represent the four treatments (FM, CK, AL, and AH). The color bar on the right indicates genus abundance (0 to 4), with blue for low abundance and red for high abundance. The colored bar on the left indicates the family-level taxonomy of each genus, as shown in the taxonomy legend. Hierarchical clustering was performed using Euclidean distance and Ward’s linkage method. The heatmap was generated using the pheatmap package in R. FM, fresh material before ensiling; CK, control (no apple pomace addition); AL, low apple pomace addition (5% fresh weight of alfalfa); AH, high apple pomace addition (10% fresh weight).

**Figure 7:**
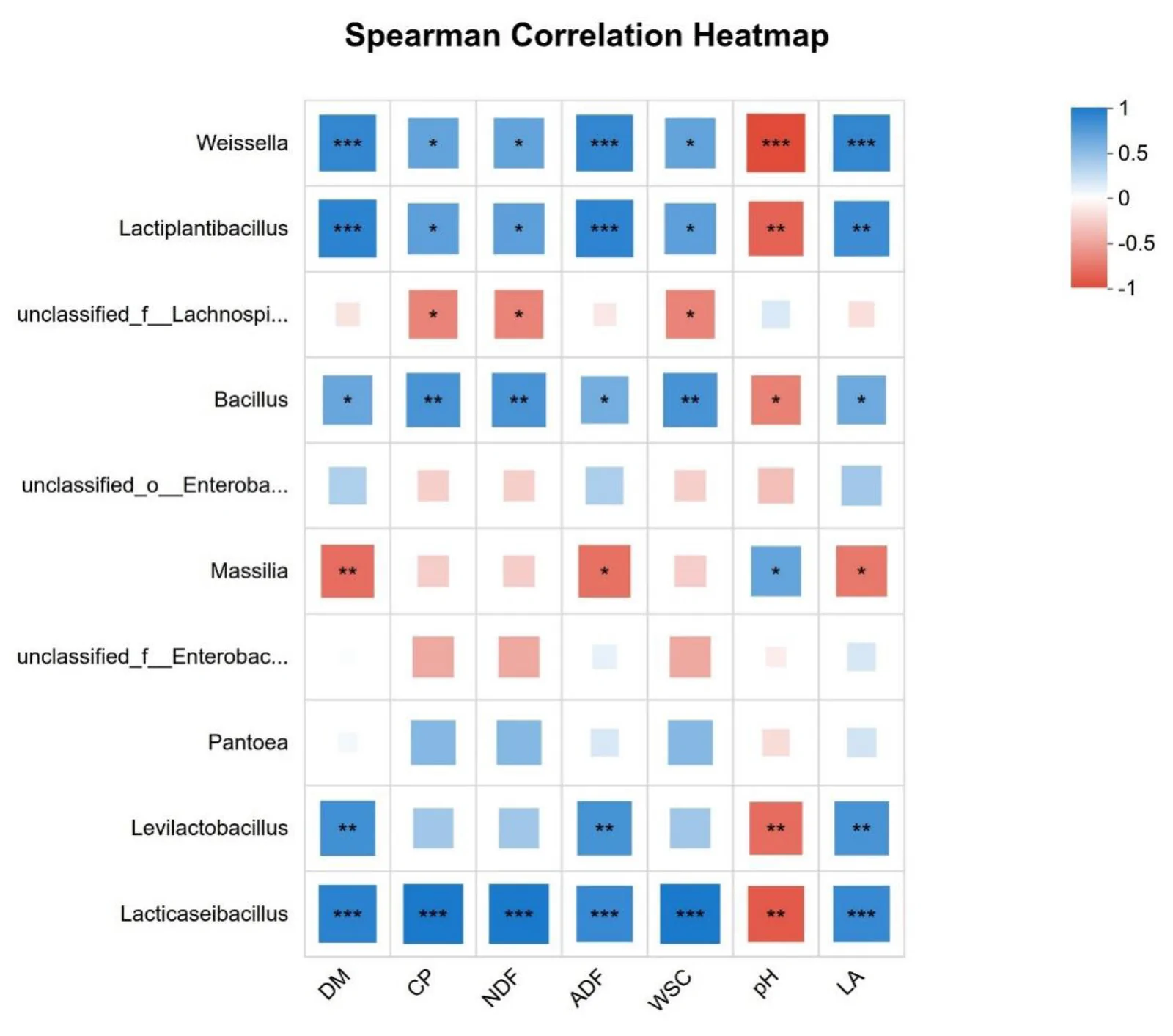
Heatmap of correlations among microbial taxa. Spearman correlation heatmap between core microbial genera and silage physicochemical parameters. Correlation coefficients (r) are color-coded from blue (negative correlation) to red (positive correlation). Physicochemical parameters include pH, lactic acid (LA), dry matter (DM), crude protein (CP), water-soluble carbohydrates (WSC), neutral detergent fiber (NDF), and acid detergent fiber (ADF). Only genera with mean relative abundance >1% are shown. Spearman rank correlation and significance tests were performed using R with the corrplot package. * p<0.05; ** p<0.01; *** p<0.001.

**Figure 8:**
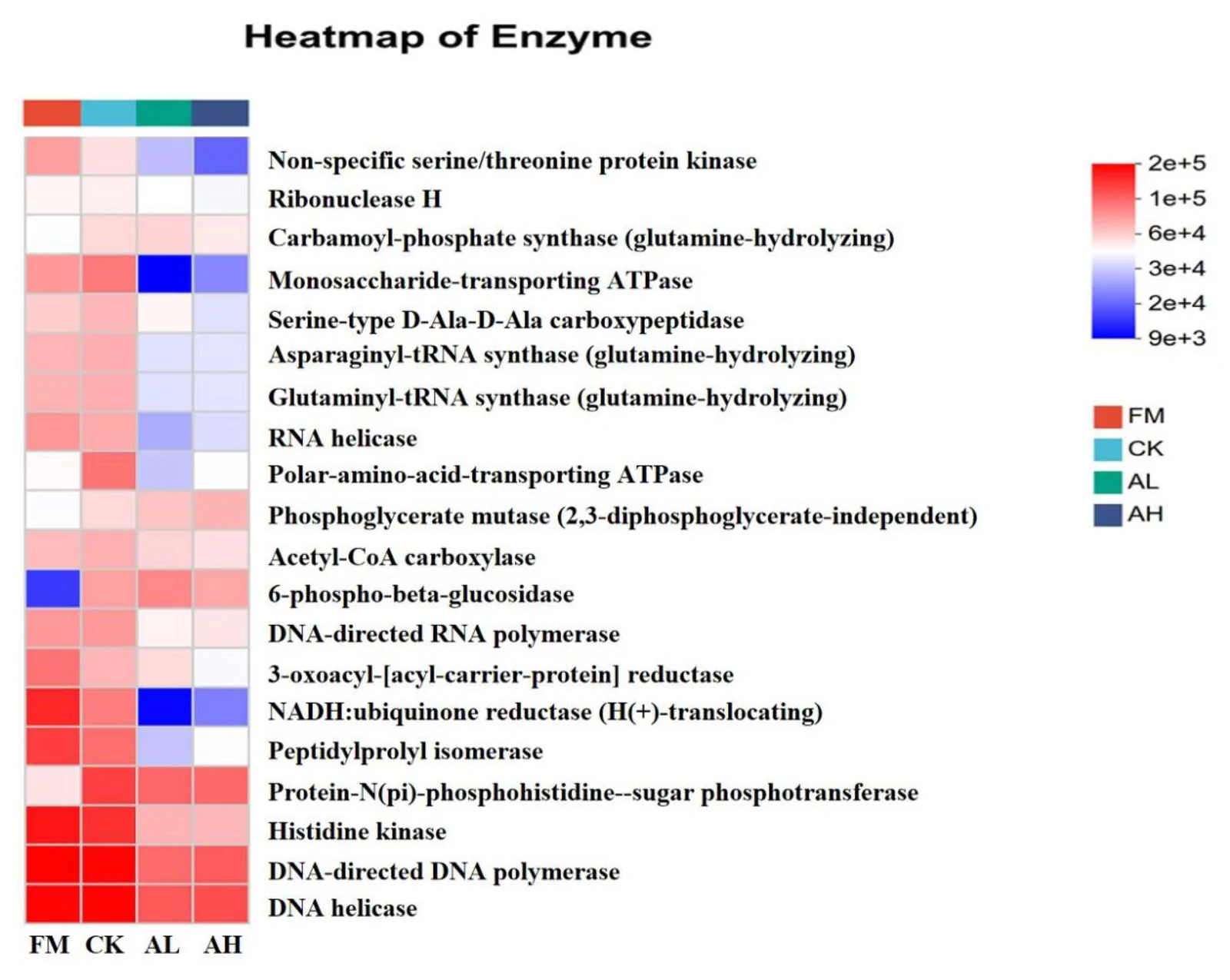
Heatmap of correlations between microbes and enzyme activities. Enzyme profiles were predicted from 16S rRNA gene sequences using PICRUSt2. The heatmap shows the relative abundance of KEGG orthologs (KO) related to carbohydrate degradation, energy metabolism, and substrate transport (e.g., 6-phospho-β-glucosidase, acetyl-CoA carboxylase) across FM, CK, AL, and AH groups. Rows represent individual enzymes, columns represent samples. Color scale (blue to red) indicates normalized enzyme abundance (Z-score). Hierarchical clustering was performed using Euclidean distance. Groups are indicated by colored bars: FM (red), CK (cyan), AL (green), AH (dark blue). Differences in enzyme abundance were tested using Welch’s t-test (p<0.05) between groups. FM, fresh material before ensiling; CK, control (no apple pomace addition); AL, low apple pomace addition (5% fresh weight of alfalfa); AH, high apple pomace addition (10% fresh weight).

**Table 1:** Proximate chemical composition of raw materials

Items	Alfalfa	Apple pomace	SEM
DM (g/kg FM)	357	425	2.93
Crude protein (g/kg DM)	174	52.9	1.59
Acid detergent fiber (g/kg DM)	392	336	2.72
Neutral detergent fiber (g/kg DM)	478	425	2.55
Water soluble carbohydrates (g/kg DM)	37.5	45.1	0.40
pH	5.37	4.94	0.03

DM, dry matter; FM, fresh material; SEM, standard error of the mean.

**Table 2:** Changes in chemical composition of alfalfa silage during ensiling

Items	CK	AL	AH	SEM	p-value	

					L	Q
DM (g/kg FW)	358^b^	436^a^	498^a^	0.383	<0.0001	0.004
Crude protein (g/kg DM)	154^b^	173^a^	193^a^	0.119	<0.0001	0.981
Acid detergent fiber (g/kg DM)	359^b^	384^a^	423^a^	0.182	<0.0001	0.045
Neutral detergent fiber (g/kg DM)	443^b^	495^a^	538^a^	0.271	<0.0001	0.011
Water soluble carbohydrates (g/kg DM)	45.1^b^	51.2^a^	57.7^a^	0.025	0.002	0.156

a,b Lowercase letters indicate the difference under different additives.

CK, no additive control; AL, low apple pomace addition (5% based on fresh alfalfa weight); AH, high apple pomace addition (10% based on fresh alfalfa weight); SEM, standard error of the mean; DM, dry matter; FM, fresh material.

**Table 3:** Fermentation dynamics of alfalfa silage with apple pomace addition

Items	CK	AL	AH	SEM	p-value	

					L	Q
pH	4.81^a^	4.12^b^	3.75^b^	0.023	<0.0001	<0.0001
Lactic acid (g/kg DM)	15.2^b^	25.7^a^	28.4^a^	0.335	<0.0001	<0.0001
Acetic acid (g/kg DM)	2.33^b^	5.12^a^	6.87^a^	0.012	<0.0001	0.003
Propionic acid (g/kg DM)	ND	ND	ND	-	-	-
Butyric acid (g/kg DM)	ND	ND	ND	-	-	-
NH_3_-N (g/kg TN)	70.3^a^	42.9^b^	39.0^b^	0.947	<0.0001	<0.0001

a,b Lowercase letters indicate the difference under different additives.

CK, no additive control; AL, low apple pomace addition (5% based on fresh alfalfa weight); AH, high apple pomace addition (10% based on fresh alfalfa weight); SEM, standard error of the mean; DM, dry matter; ND, not detected; NH_3_-N, ammonia nitrogen; TN, total nitrogen.

## Data Availability

Upon reasonable request, the datasets of this study can be available from the corresponding author.
